# Protocol for Combined Analysis of FOXFIRE, SIRFLOX, and FOXFIRE-Global Randomized Phase III Trials of Chemotherapy +/- Selective Internal Radiation Therapy as First-Line Treatment for Patients With Metastatic Colorectal Cancer

**DOI:** 10.2196/resprot.7201

**Published:** 2017-03-28

**Authors:** Pradeep S Virdee, Joanna Moschandreas, Val Gebski, Sharon B Love, E Anne Francis, Harpreet S Wasan, Guy van Hazel, Peter Gibbs, Ricky A Sharma

**Affiliations:** ^1^ Centre for Statistics in Medicine University of Oxford Oxford United Kingdom; ^2^ NHMRC Clinical Trials Centre University of Sydney Sydney Australia; ^3^ Oncology Clinical Trials Office Department of Oncology University of Oxford Oxford United Kingdom; ^4^ Imperial College Healthcare NHS Trust Hammersmith Hospital London United Kingdom; ^5^ University of Western Australia Perth Australia; ^6^ Western Hospital Footscray Victoria Australia; ^7^ National Institute for Health Research University College London Hospitals Biomedical Research Centre London United Kingdom; ^8^ Cancer Research United Kingdom–Medical Research Council Oxford Institute for Radiation Oncology University of Oxford Oxford United Kingdom

**Keywords:** colorectal neoplasms, liver, neoplasm metastasis, radiation oncology, survival analysis, meta-analysis

## Abstract

**Background:**

In colorectal cancer (CRC), unresectable liver metastases are associated with a poor prognosis. The FOXFIRE (an open-label randomized phase III trial of 5-fluorouracil, oxaliplatin, and folinic acid +/- interventional radioembolization as first-line treatment for patients with unresectable liver-only or liver-predominant metastatic colorectal cancer), SIRFLOX (randomized comparative study of FOLFOX6m plus SIR-Spheres microspheres versus FOLFOX6m alone as first-line treatment in patients with nonresectable liver metastases from primary colorectal carcinoma), and FOXFIRE-Global (assessment of overall survival of FOLFOX6m plus SIR-Spheres microspheres versus FOLFOX6m alone as first-line treatment in patients with nonresectable liver metastases from primary colorectal carcinoma in a randomized clinical study) clinical trials were designed to evaluate the efficacy and safety of combining first-line chemotherapy with selective internal radiation therapy (SIRT) using yttrium-90 resin microspheres, also called transarterial radioembolization.

**Objective:**

The aim of this analysis is to prospectively combine clinical data from 3 trials to allow adequate power to evaluate the impact of chemotherapy with SIRT on overall survival.

**Methods:**

Eligible patients are adults with histologically confirmed CRC and unequivocal evidence of liver metastases which are not treatable by surgical resection or local ablation with curative intent at the time of study entry. Patients may also have limited extrahepatic metastases. Final analysis will take place when all participants have been followed up for a minimum of 2 years.

**Results:**

Efficacy and safety estimates derived using individual participant data (IPD) from SIRFLOX, FOXFIRE, and FOXFIRE-Global will be pooled using 2-stage prospective meta-analysis. Secondary outcome measures include progression-free survival (PFS), liver-specific PFS, health-related quality of life, response rate, resection rate, and adverse event profile. The large study population will facilitate comparisons of low frequency adverse events and allow for more robust safety analyses. The potential treatment benefit in those patients who present with disease confined to the liver will be investigated using 1-stage IPD meta-analysis. Efficacy will be analyzed on an intention-to-treat basis.

**Conclusions:**

This analysis will assess the impact of SIRT combined with chemotherapy on overall survival in the first-line treatment of metastatic CRC. If positive, the results will change the standard of care for this disease.

**Trial Registration:**

FOXFIRE ISRCTN Registry ISRCTN83867919; http://www.isrctn.com/ISRCTN83867919 (Archived by WebCite at http://www.webcitation.org/6oN7axrvA). SIRFLOX ClinicalTrials.gov NCT00724503; https://clinicaltrials.gov/ ct2/show/NCT00724503 (Archived by WebCite at http://www.webcitation.org/6oN7lEGbD). FOXFIRE-Global ClinicalTrials.gov NCT01721954; https://clinicaltrials.gov/ct2/show/NCT01721954 (Archived by WebCite at http://www.webcitation.org/ 6oN7vvQvG).

## Introduction

The 5-year overall survival (OS) of metastatic colorectal cancer (mCRC) patients, who constitute 21% of all CRC diagnoses, is approximately 13% [1]. The liver is the dominant site of metastases in CRC; liver metastases are the most common cause of death for patients with CRC [2,3]. To improve outcomes in mCRC, efforts have been made to increase the proportion of patients eligible for surgical resection, which is currently 20% [4-8]. The use of down-staging neoadjuvant chemotherapy in clinical studies has suggested that 10%-20% of patients with inoperable liver disease may be converted to candidates for curative resection [9]. Furthermore, a statistical correlation between tumor response and resection rates has been found across clinical studies [10].

Among the liver-directed therapies that may control liver metastases, selective internal radiation therapy (SIRT) is one option for patients with liver-only or liver-dominant disease [11,12]. SIR-Spheres (Sirtex Medical Limited) containing the β-emitter, yttrium-90, are delivered into the arterial supply of the liver under fluoroscopic guidance. The delivery of the resin microspheres into branches of the hepatic artery, which supplies the majority of blood to liver tumors, results in selective targeting of the tumor by high-dose radiotherapy, whereas the healthy liver is supplied predominantly by the portal venous system and is therefore relatively spared from radiation treatment [12].

The FOXFIRE (an open-label randomized phase III trial of 5-fluorouracil, oxaliplatin, and folinic acid +/- interventional radioembolization as first-line treatment for patients with unresectable liver-only or liver-predominant metastatic colorectal cancer), SIRFLOX (randomized comparative study of FOLFOX6m plus SIR-Spheres microspheres versus FOLFOX6m alone as first-line treatment in patients with nonresectable liver metastases from primary colorectal carcinoma), and FOXFIRE-Global (assessment of overall survival of FOLFOX6m plus SIR-Spheres microspheres versus FOLFOX6m alone as first-line treatment in patients with nonresectable liver metastases from primary colorectal carcinoma in a randomized clinical study) clinical trials were designed to study SIRT in combination with chemotherapy, specifically the modified fluorouracil, leucovorin, and oxaliplatin (FOLFOX) regimen, compared with FOLFOX alone as first-line therapy for mCRC [13,14]. Eligibility criteria and trial designs were similar for the 3 trials so that they could be prospectively combined. The primary and some secondary endpoints of SIRFLOX have been published, representing the largest published, randomized, multicenter trial of any liver-directed therapy in patients with mCRC [15]. The results showed increased progression-free survival (PFS) in the liver with the addition of SIRT but no effect of SIRT on overall PFS [15].

The primary objective of the combined analysis is to report the planned meta-analysis (MA) of individual participant data (IPD) from the FOXFIRE, SIRFLOX, and FOXFIRE-Global studies for the primary endpoint, OS, and for secondary outcomes including PFS, liver-specific PFS, quality of life measures, response rate, resection rate, and adverse event profile. The IPD-MA will assess the treatment effects on clinical outcomes in the entire subject group and in key subgroups.

## Methods

### Study Design

As the results on survival benefits for each of the 3 studies are still blinded, the statistical design is a prospective MA based on randomized IPD from the FOXFIRE, SIRFLOX, and FOXFIRE-Global clinical trials.

**Figure 1:**
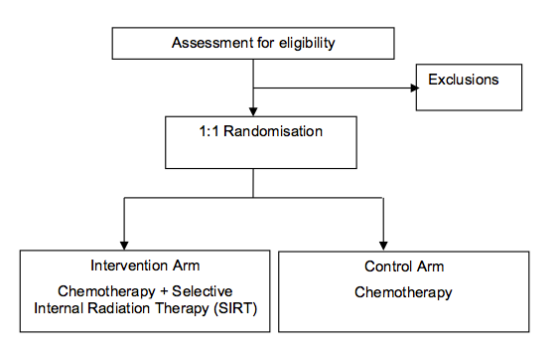
Schema for the FOXFIRE, SIRFLOX, and FOXFIRE-Global trials.

The protocols of the FOXFIRE [13] and SIRFLOX [14] trials have been previously published. All 3 clinical trials were open-label multicenter, randomized, 2-arm trials comparing chemotherapy plus SIRT with chemotherapy alone as first-line treatment for patients with mCRC. The design of the 3 trials is summarized in Table 1 and Figure 1.

**Table 1:** Characteristics of studies included in the meta-analysis: FOXFIRE [13], SIRFLOX [14], and FOXFIRE-Global.

	FOXFIRE^a^	SIRFLOX^b^	FOXFIRE-Global^c^
Start of recruitment	Nov 13, 2009	Oct 11, 2006	May 20, 2013
End of recruitment	Oct 31, 2014	Apr 25, 2013	Dec 23, 2014
End of follow-up	Oct 31, 2016	Apr 25, 2018	Dec 23, 2019
Number of recruiting centers	28	87	69
Primary objective	Overall survival	Progression-free survival	Overall survival
Secondary objectives	Progression-free survival Progression-free survival in the liver Toxicity and safety Tumor response rate Quality of life Liver resection rate Health costs/economics Proportion of patients receiving second line treatment Time to second line treatment	Overall survival Progression-free survival in the liver Toxicity and safety Tumor response rate Quality of life Liver resection rate Hepatic and extrahepatic recurrence rate	Progression-free survival Progression-free survival in the liver Toxicity and safety Tumor response rate Quality of life Liver resection rate Hepatic and extrahepatic recurrence rate Health economics
Sample size accrued	364	530	209
Accrual period	November 2009–October 2014	October 2006–April 2013	May 2013–December 2014
Follow-up period	November 2014–October 2016	April 2013–April 2018	December 2014–December 2019
Randomization	1:1 with minimization	1:1 with minimization	1:1 with minimization
Minimization factors	Liver only versus extrahepatic metastases Extent of tumor involvement of the liver (≤25% or >25% determined by CT^d^ scan) Planned use of biological agent (from March 2011 on) Investigational center	Liver only versus extrahepatic metastases Extent of tumor involvement of the liver (≤25% or >25% determined by CT scan) and based upon the tumor involvement groupings used by Gray et al [16] Planned use of bevacizumab with chemotherapy Investigational center	Liver only versus extrahepatic metastases Extent of tumor involvement of the liver (≤25% or >25% determined by CT scan) and based upon the tumor involvement groupings used by Gray et al [16] Planned use of bevacizumab with chemotherapy Investigational center
Recruiting countries/regions	United Kingdom (England, Northern Ireland, Scotland, Wales)	Australia, Europe, Israel, New Zealand, and United States	Australia, Europe, Israel, Korea, New Zealand, Singapore, Taiwan and United States

^a^An open-label randomized phase III trial of 5-fluorouracil, oxaliplatin, and folinic acid +/- interventional radioembolization as first-line treatment for patients with unresectable liver-only or liver-predominant metastatic colorectal cancer.

^b^Randomized comparative study of FOLFOX6m plus SIR-Spheres microspheres versus FOLFOX6m alone as first-line treatment in patients with nonresectable liver metastases from primary colorectal carcinoma.

^c^Assessment of overall survival of FOLFOX6m plus SIR-Spheres microspheres versus FOLFOX6m alone as first-line treatment in patients with nonresectable liver metastases from primary colorectal carcinoma in a randomized clinical study.

^d^Computed tomography.

### Patients and Treatment

All randomized patients from the SIRFLOX, FOXFIRE, and FOXFIRE-Global clinical trials will be included in the combined analysis.

#### Inclusion Criteria

- Histologically confirmed CRC with liver-only or liver-dominant metastases not amenable to surgical resection, primary tumour in situ permitted (FOXFIRE); histologically confirmed CRC with liver-only or liver-dominant metastases (SIRFLOX); histologically confirmed adenocarcinoma of the colon or rectum with or without primary tumor in situ (FOXFIRE-Global)
- Unequivocal and measurable computed tomography (CT) evidence of liver metastases not treatable by surgical resection or local ablation with curative intent at time of trial entry
- Chemotherapy-naïve for mCRC, but previous adjuvant systemic chemotherapy for primary CRC or neoadjuvant chemoradiotherapy to the pelvis more than 6 months before recruitment is permitted (SIRFLOX, FOXFIRE-Global); eligible for systemic chemotherapy as first-line treatment for metastatic CRC (FOXFIRE)
- Additional limited extrahepatic metastases in the lung or lymph nodes permitted (SIRFLOX, FOXFIRE-Global); patients are permitted to have limited extrahepatic disease (FOXFIRE)
- Age ≥18 years
- World Health Organization performance status of 0-1
- Life expectancy >3 months
- Adequate hematological, renal, and hepatic function
- Female patients must be postmenopausal or using adequate contraception if premenopausal, and male patients must be using an appropriate method of contraception if with a premenopausal partner (FOXFIRE); female patients must either be postmenopausal, sterile (surgically or chemically or radiation-induced) or if sexually active using an acceptable method of contraception, and male patients must be surgically sterile or if sexually active and have a premenopausal partner must be using an acceptable method of contraception (SIRFLOX, FOXFIRE-Global)
- Suitable for all aspects of treatment determined by clinical assessment undertaken by investigator
- Willing and able to provide written informed consent

FOXFIRE-Global and SIRFLOX:

- All imaging evidence used as part of the screening process must be within 28 days prior to randomization

#### Exclusion Criteria

- Evidence of ascites, cirrhosis, portal hypertension; tumour involvement of, or thrombosis leading to occlusion of the main portal vein
- Previous radiotherapy delivered to upper abdomen or upper lumbar spine (FOXFIRE); previous radiotherapy delivered to the upper abdomen (SIRFLOX); previous radiotherapy delivered to the liver (FOXFIRE-Global)
- Nonmalignant disease that would render patient ineligible at the discretion of the investigator
- Dose-limiting toxicity associated with previous 5-fluorouracil or oxaliplatin chemotherapy
- Peripheral neuropathy higher than grade 1 (National Cancer Institute–Common Terminology Criteria for Adverse Events [NCI-CTCAE] version 3)
- Pregnant or breastfeeding
- Previous chemotherapy for any malignancy; adjuvant chemotherapy for colorectal cancer is not an exclusion criterion provided that it was completed more than 6 months prior to entry into the study (SIRFLOX, FOXFIRE-Global). Previous chemotherapy for metastatic colorectal cancer; adjuvant chemotherapy for colorectal cancer is not an exclusion criterion provided that it was completed more than 6 months prior to entry into the study (FOXFIRE)
- Concurrent or prior history of cancer other than adequately treated nonmelanoma skin cancer or carcinoma in situ of the cervix (SIRFLOX, FOXFIRE-Global); other active malignancy within last 5 years excluding colorectal cancer and other nonmelanoma skin cancers (FOXFIRE)

SIRFLOX and FOXFIRE-Global:

Allergy to nonionic contrast agents

FOXFIRE only:

- Liver metastases amenable to curative resection at time of study entry
- Equivocal, immeasurable, or nonevaluable liver metastases
- Unequivocal evidence of bone metastasis

All patients received first-line chemotherapy for mCRC. In FOXFIRE, the chemotherapy received was oxaliplatin, 5-fluorouracil, and leucovorin/folic acid in the OxMdG regime [17]. In SIRFLOX and FOXFIRE-Global, the chemotherapy consisted of the same drugs in the mFOLFOX6 regime [18]. Patients were randomized 1:1 to the addition of SIRT using yttrium-90 resin microspheres, using minimization. The minimization factors are given in Table 1. Biological agents (cetuximab or bevacizumab in FOXFIRE; bevacizumab in SIRFLOX) were permitted to be added to chemotherapy at the treating investigators’ discretion. Protocol treatment in each study was commenced within 28 days of randomization. Further details on randomization by minimization and treatment regimens for FOXFIRE and SIRFLOX have been published previously [13,14]. FOXFIRE-Global used the same randomization method and treatment regimen as that used for the SIRFLOX study.

In all 3 trials, patients were assessed every 2 weeks during chemotherapy treatment. In SIRFLOX and FOXFIRE-Global, patients were assessed every 12 weeks during the postprogression follow-up period. FOXFIRE patients were assessed every 8 weeks following completion of treatment up to 18 months and then every 12 weeks thereafter. In all 3 trials, patients were followed until death or for a period of at least 2 years after randomization. Patients undergoing surgical resection after trial entry were also followed up until trial closure or until death.

Screening and follow-up assessments included clinical assessment and laboratory analyses, recording concurrent medications, CT scan of the chest/abdomen/pelvis with or without magnetic resonance imaging, recording adverse events, assessment for resection or ablation, and questionnaire-based assessments of quality of life.

### Study Outcomes

#### Primary Outcome of the Combined Analysis

The primary outcome of OS is the time from randomization to death from any cause, with patients still alive being censored at their last known follow-up date.

#### Secondary Outcomes of the Combined Analysis

The secondary outcomes are PFS at any site, liver-specific PFS, health-related quality of life (HRQoL), response rate, resection rate, and the safety profiles. PFS is defined as the time from randomization to radiological progression according to Response Evaluation Criteria In Solid Tumors (RECIST version 1.0) or death from any cause, whichever is sooner. Patients who are not observed to progress or die during the course of the trial will be censored at last known progression-free follow-up date. Patients who withdraw from study treatment prior to documented progression will be censored at the time they commence nonstudy treatment. Patients who become eligible for resection will be considered as still being on study until progression is documented or last follow-up. Scans and tumor response will be centrally reviewed in FOXFIRE and SIRFLOX.

Liver-specific PFS is defined as the time from randomization to radiological progression in the liver (hepatic progression) according to RECIST version 1.0. Progression outside the liver (extrahepatic progression) and deaths prior to progression will be considered as competing risks for failure in the liver. Hepatic progression will be assumed to have occurred immediately before extrahepatic progression in patients who have identical hepatic and extrahepatic progression dates. Patients who withdraw from study treatment prior to any documented progression will be censored at the time they commence nonstudy treatment. Patients who become eligible for resection will be considered as still being on study until progression is documented or last follow-up.

HRQoL will be assessed using the EuroQol 5 dimensions questionnaire (EQ-5D). The EQ-5D comprises the following 5 dimensions: mobility, self-care, usual activities, pain/discomfort, and anxiety/depression. Each of the 5 dimensions is scored, generating a profile. A single index score or utility value, representing population-derived preferences for different health states, will be attached to each profile.

The response rate (objective response) is defined as the number of patients achieving a complete or partial response according to RECIST version 1.0 divided by the number of patients in each treatment arm. Early death by any cause and unknown responses will be included in the denominator. Response will be assessed over 2 time periods: within 12 months of randomization and over follow-up.

The resection rate in each treatment arm is defined as the number of patients undergoing resection of their liver metastases divided by the number of patients randomized in each arm. Patients undergoing treatment are assessed by an experienced liver multidisciplinary team or equivalent for eligibility for resection at the discretion of the treating physician.

The safety profile is assessed by the collection of adverse events (AEs) and serious adverse events (SAEs) at any time during treatment with grading using the NCI-CTCAE version 3.

### Sample Size Calculation

For the primary combined OS analysis, the FOXFIRE study protocol details a calculated 1075 patients required using a protocol-specified hazard ratio (HR) of 0.8, a 36-month accrual period, and 18-month minimum follow-up with 2-sided 5% significance, 80% power, and allowing for noncompliance. A total of 710 deaths are expected. The required sample size had been previously calculated to be 1022 [13] but was updated to reflect an estimated increase in median OS in the control group. As the intervention is a local treatment to the liver only, it is anticipated that even if there is no OS benefit in the whole population, a demonstrated survival benefit in the liver-only subgroup would be clinically meaningful and could change practice. It is anticipated that, with a 6-month increase in OS in the SIRT treated liver-only patients compared to those randomized to chemotherapy only, this would require 463 events in the liver-only subgroup.

### Closure of Study

The final analysis will be undertaken when a minimum of 710 deaths overall and 463 deaths in the liver-only subgroup have been observed in the pooled dataset and there has been a minimum follow-up of 2 years since the last patient was randomized into the 3 trials.

### Data Monitoring Committee and Interim Analyses

The independent data and safety monitoring committee (IDSMC) consisted of the same representatives for the FOXFIRE, SIRFLOX, and FOXFIRE-Global clinical trials. Formal interim monitoring of the accumulating data was performed at regular intervals (approximately every 6 months) by the IDSMC for each trial separately. This information included results from other relevant trials but not the analysis of primary or secondary objectives or outcomes by treatment groups apart from the prespecified interim analyses. As part of the review, the IDSMC was asked to justify continued recruitment of further patients or further follow-up. The IDSMC advised on the frequency of future reviews of the data on the basis of accrual and event rates.

The IDSMC reviewed the combined safety data from FOXFIRE and SIRFLOX at the interim analyses. The following planned interim analyses were undertaken using combined data from the FOXFIRE and SIRFLOX trials:

- Analysis of toxicity and safety: 8 months after at least 80 patients were randomized in total (a minimum of 40 per trial)
- Analysis of toxicity and safety: 8 months after at least 300 patients were randomized in total (a minimum of 120 patients per trial)

The 3 clinical trials that constitute this MA were conducted in accordance with the Declaration of Helsinki and current Good Clinical Practice guidelines, and all participating centers obtained the relevant ethics committee approval before patient enrollment. FOXFIRE was approved by the National Research Ethics Service Committee South Central – Berkshire Research Ethics Committee reference 09/H0505/1 and sponsored by the University of Oxford. SIRFLOX and FOXFIRE-Global were approved by the relevant ethics committees for each center and sponsored by Sirtex Technology Pty Ltd.

### Statistical Analysis

#### Descriptive Summaries

Summaries of baseline factors, including minimization factors, and percentages of missing data will be reported. Losses to follow-up will be reported for each trial and combined. Median follow-up time will be calculated using the reverse Kaplan-Meier method. Dose of trial-specific treatment delivered and treatment received subsequent-to-trial treatment will be described.

#### Efficacy

All efficacy analyses will be performed on an intention-to-treat (ITT) basis. OS and PFS estimates will be obtained using Kaplan-Meier survival curves, unadjusted log-rank tests, and survival models. A 2-stage inverse-variance weighted IPD-MA will be performed for both OS and PFS, with the first stage consisting of trial-specific analyses to obtain efficacy estimates (HRs) and the second stage being a pooled analysis of the separate trial-specific HRs. As a sensitivity analysis, a 1-stage IPD-MA using regression models stratified by study will be performed to confirm the results obtained from the 2-stage IPD-MA. For each outcome, multivariable models will be used in the 1-stage IPD-MA to account for baseline covariates.

Liver-specific PFS will be analyzed using cumulative incidence curves, Gray’s test, and competing risks regression. This analysis will be performed on the pooled dataset with models stratified by trial. For OS, PFS, and liver-specific PFS, the potential treatment benefit in those patients who present with disease confined to the liver will be investigated. This prespecified subgroup analysis will be performed on the pooled dataset. The analysis strategy will include calculating HR for the effect of treatment in the liver-only subgroup using survival models stratified by trial.

A separate landmark analysis of OS will start at the 15-month time point (after randomization) and therefore exclude those who have died/withdrawn within 15 months of randomization. This time point has previously been considered of value in clinical trials of the treatment of mCRC [19]. Based on expert opinion, it is anticipated that 15 months from baseline allows sufficient time for events to occur in those with both diagnosed extrahepatic metastases and subclinical extrahepatic metastases at baseline, and therefore this analysis may be of value in detecting any differential survival impact of SIRT in patients with liver-only metastases.

The EQ-5D data will be merged across all 3 trials and summary data prepared on the mean EQ-5D utility score in each trial arm by time period, with appropriate tests for difference. Resection rate and response rate will be analyzed using chi-square tests and odds ratios (ORs) for the individual trials and a 2-stage IPD-MA.

#### Safety

Safety analyses will be performed on patients who received at least 1 dose of chemotherapy in either arm and on an as-treated basis (safety population). AEs experienced up to 28 days after the end of trial treatment or 7 months postrandomization, whichever was earlier, will be included. Descriptive summaries of the frequency and severity of AEs and the numbers of patients experiencing AEs of grade 3 or higher between treatment arms will be presented overall and by system organ class. The Medical Dictionary for Regulatory Activities version 16.1 is used to categorize the AEs. Univariate comparisons will be made.

All hypothesis tests will be 2-sided. A significance level of .05 will be used to indicate statistical significance. No missing data imputation is intended. Missing days in dates may be appropriately imputed.

## Results

Data from the final data lock will become available in January 2017. Data analysis will take place in 2017 with results being disseminated via peer-reviewed journals in 2017 and 2018.

## Discussion

Optimizing outcomes of treatment in patients with nonresectable liver metastases was identified in an international consensus expert statement as a key clinical need to be addressed [20]. This is the first extensive investigation of SIRT in the first-line setting for liver metastases from mCRC adequately powered to address an overall survival endpoint.

This prospective MA of 3 phase III studies will provide comprehensive evidence of the safety and potential efficacy of SIRT in the first-line setting for patients with liver metastases from mCRC. The results of the SIRFLOX trial published so far have suggested that the addition of SIRT to chemotherapy can improve liver-specific PFS [15]. The number of patients included in this combined analysis and the long-term follow-up, unprecedented in the field of interventional oncology, will provide adequate power to address a survival endpoint.

Although the SIRFLOX trial reported that liver-specific PFS was longer in the SIRT arm than in the control arm, the PFS at any site was similar between the SIRT arm and the control arm [15]. This can be accounted for by the fact that SIRT is a locoregional treatment to the liver only and therefore will not influence the progression of extrahepatic metastases or extrahepatic subclinical micrometastases.

It is generally accepted that successful resection of liver metastases correlates with improved overall survival, particularly in the context of neoadjuvant or adjuvant chemotherapy [21,22]. Surgery and perioperative chemotherapy are therefore routinely offered to patients with mCRC if the liver metastases are resectable. Similarly, the addition of radiofrequency ablation of liver metastases to standard chemotherapy appears to improve clinical outcomes for patients compared to those patients receiving standard chemotherapy alone [23,24]. These findings with liver-directed therapies suggest that improvement of liver-specific PFS in patients with liver metastases may correlate to improvement in OS, an important hypothesis to be tested for SIRT for the first time in this combined MA of the SIRFLOX, FOXFIRE, and FOXFIRE-Global trials.

The use of an IPD rather than an aggregate data approach to systematic review and MA of randomized controlled trials enables the standardization of outcomes across trials and detailed data checking, providing a more in-depth exploration and more robust MA results [25]. The proposed MA, by prospectively combining IPD from 3 trials, goes beyond classical MA aims and overrides drawbacks from single trials. The prospective approach allows for consistent inclusion and exclusion criteria between studies. The statistical analysis is going to be standardized across studies, and using IPD-MA will provide sufficient statistical power to draw conclusions from subgroup analyses that are generally undermined by low sample size. The use of the 2-stage approach for the main analyses of OS and PFS is prespecified, as the 2-stage approach might produce different parameter estimates to the 1-stage approach, although the estimates are usually similar [26,27].

The results from the combined analysis of the SIRFLOX, FOXFIRE, and FOXFIRE-Global trials will provide valuable clinical information on the efficacy and toxicity profile of SIRT combined with chemotherapy as a first-line regimen for liver metastases from mCRC that will guide clinicians in the use of this technology to treat their patients. The IPD-MA will allow comparisons of less common AEs that would not be possible in a smaller population. Furthermore, this prospective MA provides sufficient power to determine whether SIRT provides a significant survival benefit for patients with metastases confined to the liver and no clinically detectable extrahepatic disease, an important research question among clinicians treating mCRC. When reported, the results of this combined analysis will define the use of SIRT in the treatment of mCRC.

## Abbreviations

AE: adverse event
CRC: colorectal cancer
CT: computed tomography
EQ-5D: EuroQol 5 dimensions questionnaire
FOLFOX: modified fluorouracil, leucovorin, and oxaliplatin regimen
FOXFIRE: An open-label randomized phase III trial of 5-fluorouracil, oxaliplatin, and folinic acid +/- interventional radioembolization as first-line treatment for patients with unresectable liver-only or liver-predominant metastatic colorectal cancer
FOXFIRE-Global: Assessment of overall survival of FOLFOX6m plus SIR-Spheres microspheres versus FOLFOX6m alone as first-line treatment in patients with nonresectable liver metastases from primary colorectal carcinoma in a randomized clinical study
HR: hazard ratio
HRQoL: health-related quality of life
IDSMC: independent data and safety monitoring committee
IPD: individual participant data
IPD-MA: Individual participant data meta-analysis
ITT: intention-to-treat
MA: meta-analysis
mCRC: metastatic colorectal cancer
NCI-CTCAE: National Cancer Institute–Common Terminology Criteria for Adverse Events
OR: odds ratio
OS: overall survival
PFS: progression-free survival
RECIST: Response Evaluation Criteria In Solid Tumors
SAE: serious adverse events
SIRFLOX: Randomized comparative study of FOLFOX6m plus SIR-Spheres microspheres versus FOLFOX6m alone as first-line treatment in patients with nonresectable liver metastases from primary colorectal carcinoma
SIRT: selective internal radiation therapy
